# Main Recommendations for Developing Education and Awareness Strategies for Rare Diseases: Scoping Review

**DOI:** 10.2196/79027

**Published:** 2026-06-12

**Authors:** Diego Bettiol Yamada, Tatiana Takahasi Komoto, Vinícius Costa Lima, Julio Souza, Michele de Souza Seixas, Victor Cassão, Victor Evangelista de Faria Ferraz, Bibiana Mello de Oliveira, Têmis Maria Félix, Domingos Alves

**Affiliations:** 1 Ribeirao Preto Medical School, University of Sao Paulo Ribeirao Preto Brazil; 2 RISE-Health, Faculty of Medicine, University of Porto Porto Portugal; 3 Institute of Engineering, Polytechnic of Porto Porto Portugal; 4 Federal University of Health Sciences of Porto Alegre Porto Alegre Brazil; 5 Medical Genetics Service, Hospital de Clinicas de Porto Alegre Porto Alegre Brazil; 6 Brazilian National Institute on Rare Diseases Porto Alegre Brazil

**Keywords:** educational strategies, health awareness, health education, public health recommendations, rare diseases

## Abstract

**Background:**

According to the World Health Organization, education and awareness are essential components of public health promotion strategies. In the context of rare diseases (RDs), these actions are particularly critical because of persistent stigma, fragmented knowledge, and the frequent absence of consolidated clinical and organizational protocols. These gaps often result in inappropriate referrals, inefficient care pathways, unnecessary procedures, and delays in diagnosis, negatively affecting health outcomes and quality of life.

**Objective:**

This study aimed to identify and systematize the main recommendations for health education and awareness in the field of RDs, supporting the development of health care programs, public policies, and strategic initiatives.

**Methods:**

We formulated the research question using the Population, Concept, and Context framework. This scoping review followed the PRISMA-ScR (Preferred Reporting Items for Systematic Reviews and Meta-Analyses Extension for Scoping Reviews) guidelines to ensure methodological transparency. Eligible records included peer-reviewed research articles of any design and official documents published in Portuguese, English, or Spanish, with no time restrictions. Records that did not address the research question, lacked sufficient rigor, or focused exclusively on specific subgroups of RDs were excluded. Searches were performed in PubMed/MEDLINE, Scopus, Embase, Web of Science, as well as gray literature. Study selection and data extraction were conducted by the research team, with disagreements resolved and the included sources reviewed by an RDs expert. Data were thematically categorized by consensus, and descriptive statistics were used to summarize findings.

**Results:**

A total of 58 sources of evidence were included. Among the identified recommendations related to education and awareness, most sources focused on professional education and training (49/58, 84.4%), followed by public policies and intersectoral integration (36/58, 62%), education and awareness for the general population (28/58, 48.2%), digital technologies (27/58, 46.5%), emotional support and experience sharing (20/58, 34.4%), and awareness events and dates (8/58, 13.7%). Percentages exceed 100% because individual sources could report multiple recommendations. Overall, the literature emphasizes integrating RDs content into educational initiatives and strengthening professional competencies, intersectoral collaboration, digital technologies, and broader awareness strategies.

**Conclusions:**

This scoping review systematically mapped and organized recommendations from diverse sources of evidence on strategies for health education and awareness related to RDs. It synthesizes heterogeneous evidence using a structured approach to provide a comprehensive overview of strategies in this field, consolidating dispersed knowledge into a coherent body of evidence. The findings may inform improvements in health services, as well as professional and managerial practices, and initiatives aimed at supporting patients, families, and advocacy groups involved in RDs, with potential implications for strengthening diagnostic processes, referral coordination, and more equitable access to information and care.

## Introduction

### Rationale

According to the World Health Organization (WHO) recommendations, education and awareness are fundamental processes for the success of public health promotion strategies [1]. In the context of rare diseases (RDs), these actions become even more necessary and relevant because of the persistence of stigma and discrimination associated with these conditions. This scenario stems from several factors, including gaps in structured and disseminated knowledge about RDs, the absence of consolidated clinical and organizational protocols within health care institutions, and the lack of coordinated frameworks capable of supporting adaptable educational initiatives [2-4]. Without shared educational approaches, public health messaging and professional training efforts often remain fragmented, limiting the dissemination of reliable information among stakeholders involved in RD care [3,4].

RDs are conditions with low prevalence in a given country or region. However, there is no global consensus on their definition, as each jurisdiction classifies them according to its own criteria. In the United States, a disease is considered rare when it affects fewer than 200,000 people or approximately 1 person per 1500 inhabitants [5]. In the European Union, a condition is classified as rare when it affects fewer than 1 person per 2000 individuals [6]. In Brazil, the Unified Health System (Portuguese: Sistema Único de Saúde) adopts the WHO criterion, which considers a disease rare if it affects up to 1.3 individuals per 2000 people [7]. Currently, there are more than 7000 known distinct RDs worldwide [8]. Although each condition individually affects a limited number of people, collectively these diseases represent a substantial public health challenge, with a global prevalence rate estimated at 5.9%, corresponding to nearly 300 million affected individuals worldwide [9,10].

The heterogeneity of RDs makes the establishment of standardized clinical protocols and care approaches particularly challenging. In addition, the lack of precise and up-to-date data regarding the incidence and prevalence of these conditions compromises health care planning and resource allocation [11]. In response to these limitations, several research and development networks focused on RDs have been established in regions such as the European Union [12], the United States [13], Brazil [3], Latin America [14], and Asia [15]. However, despite the institutionalization of these initiatives, education and awareness efforts related to RDs are not always transparent, well-structured, or effective in practice [16]. This gap in educational practices challenges health care providers, caregivers, and patients, who frequently face misinformation or limited access to reliable information, especially in regions with weaker health care systems or digital infrastructure [16,17].

Although health curricula are already overloaded, the development of engaging and innovative educational strategies for RDs remains necessary [18]. The absence of comprehensive informational and educational frameworks directly contributes to delays in diagnosis and treatment initiation within health care systems worldwide. In the United States, for instance, the average time required to diagnose a RD can reach up to 7 years, a period commonly referred to as a “diagnostic odyssey” [19,20]. This interval may be even longer in developing countries [21]. Beyond delayed diagnosis, the high informational and managerial complexity associated with RDs also contributes to referral errors, inefficient patient flows, unnecessary procedures, and inadequate care coordination. Considering that most RDs are chronic and debilitating conditions, this scenario imposes a significant burden on patients, families, health care institutions, and society as a whole [3,22,23].

In light of these considerations, effective educational and awareness strategies must be designed based on a robust understanding of existing recommendations, evidence-based practices, community experiences, and digital approaches capable of improving knowledge dissemination and promoting equity in access to care [9,16,18]. However, despite the growing recognition of the importance of education and awareness in the RD field, the available evidence remains fragmented across different disciplines, stakeholders, and health care contexts. Educational initiatives may involve health care professionals, patients, families, advocacy organizations, policymakers, and educators. This complexity highlights the need for a broad and exploratory synthesis capable of systematically mapping the literature and identifying the main recommendations regarding education and awareness in RDs.

### Objective

Thus, this study aims to conduct a scoping review to identify and systematize the main recommendations for health education and awareness in the context of RDs, supporting the development of health programs, strategies, and public policies in this area.

## Methods

### Overview

As previously mentioned, a scoping review was conducted to investigate the state of the art regarding the main global recommendations for health education and awareness in the domain of RDs. The Population, Concept, and Context (PCC) methodology was adopted to define the research question. Initially proposed by the Joanna Briggs Institute, the PCC framework is particularly robust for reviews aiming to explore and map the state of the art in the literature on a given topic, identifying key concepts, knowledge gaps, and domain-specific demands [24].

It is especially useful for exploratory themes and broad investigations. The adoption of this methodology, therefore, enables a systematic approach to conducting the stages of a scoping review, providing a solid foundation on which the study is built and contributing to more consistent and relevant results [24]. Accordingly, the guiding question of the scoping review was established and is described as follows: “What are the main recommendations on health education and awareness within the context of RDs?”

After defining the research question, the PCC framework guided the development of the search strategy. To ensure clear, systematic, and transparent reporting, this scoping review followed the PRISMA-ScR (Preferred Reporting Items for Systematic Reviews and Meta-Analyses Extension for Scoping Reviews); the completed checklist is provided in Multimedia Appendix 1. The selection of sources of evidence was illustrated using the PRISMA (Preferred Reporting Items for Systematic Reviews and Meta-Analyses) 2020 flow diagram, which can be adapted for scoping reviews and applied in conjunction with this framework. These tools are widely recognized for providing a structured approach to documenting all stages of a review, including the identification, screening, assessment of eligibility, and selection of sources, to answer the research guiding question [25,26].

The PRISMA-ScR is especially useful for documenting the aforementioned steps in a structured manner. It also serves as a support tool for research teams, reviewers, and evaluators involved in the review process. From a scientific standpoint, its use is important because it enables the reproducibility of the process by other researchers, helping to avoid redundant work while maintaining methodological rigor. For these reasons, it is considered a scientific best practice, aligned with international guidelines for conducting and reporting reviews [22,25].

In the following sections, we describe the inclusion and exclusion criteria, databases used, search strategy, study selection process, PRISMA 2020 flow diagram, and procedures for extracting knowledge from the selected sources of evidence. These procedures were designed to address the research question and guide the subsequent stages of the study.

### Protocol and Registration

A review protocol was not registered for this scoping review. The study is supported by the São Paulo Research Foundation (Fundação de Amparo à Pesquisa do Estado de São Paulo) and was approved by the Research Ethics Committee of the Hospital das Clínicas da Faculdade de Medicina de Ribeirão Preto da Universidade de São Paulo (HCFMRP-USP; protocol number CAAE 82571424.4.0000.5440).

### Eligibility Criteria

The inclusion and exclusion criteria were defined to ensure relevance, appropriateness, and precision in alignment with the investigation’s objectives. These criteria considered the types of studies, languages, publication periods, and the evaluation of the content of each source. The specific criteria are outlined in detail in Textbox 1.

**Inclusion and exclusion criteria.**

**Inclusion criteria**

- Research articles, including full papers, short communications, and peer-reviewed conference papers
- All study designs were eligible for inclusion, including qualitative, quantitative, mixed methods studies, and reviews
- Official government documents, such as policies, programs, action plans, and technical reports
- Sources published in Portuguese, English, and Spanish
- No restrictions were applied regarding the date of publication
- Sources were included if they addressed the research question and explicitly reported on relevant aspects of health education, health awareness, or literacy in the context of rare diseases (RDs)

**Exclusion criteria**

- Publications that did not meet minimum methodological standards to support the research question (eg, lacking defined methods or sources)
- Editorials, opinion pieces, and content from social media
- Sources published in languages other than those listed in the inclusion criteria
- Sources that mention relevant terms without providing substantive description or analysis of these topics
- Sources focused exclusively on specific clinical procedures, an individual RD, or specific groups of RDs, without addressing broader educational or awareness-related aspects

Given the interdisciplinary and multifaceted nature of education and awareness in the field of RDs, the inclusion criteria encompassed a broad range of study types, including qualitative, quantitative, mixed methods research, reviews, and official government documents. The inclusion of sources in English, Spanish, and Portuguese was intended to capture diverse regional perspectives, particularly from countries with established RD frameworks in the Americas and Europe, while acknowledging the central role of English in international scientific communication.

No date restrictions were applied to allow the mapping of historical and recent developments in the field. Exclusion criteria were applied to ensure conceptual and methodological consistency by filtering out documents lacking analytical depth, empirical grounding, or alignment with the scope of the review. Publications focusing narrowly on clinical aspects without addressing educational or awareness-related dimensions were excluded to maintain focus on the broader systemic and communicative strategies relevant to the objectives of the review.

### Information Sources

The databases were selected based on their scope and relevance to the research topic, literature coverage, accessibility, robustness, and the quality of the indexed studies. Therefore, the databases defined for the search were PubMed/MEDLINE, Scopus, Embase, and Web of Science.

The selection of these databases aims to ensure adequate coverage of the central theme of the review and the necessary geographic scope, while also considering the types of documents indexed, the curation of records, scientific relevance, reproducibility of results, and methodological rigor associated with validated evidence. Thus, we built and applied the search strategy within these databases to identify the studies composing the scoping review. The initial search was conducted on May 12, 2025. The search strategy was updated iteratively during the review process and was last rerun on March 15, 2026, across all databases.

In addition to bibliographic databases, gray literature was searched across governmental and institutional websites (eg, WHO, the European Commission, the US Food and Drug Administration, Rare Diseases International, and Asia-Pacific Economic Cooperation resources), as well as through Google Scholar as a supplementary web search tool. Records identified through this approach were considered eligible if they met the predefined eligibility criteria and addressed the research question. These online resources were manually browsed and searched to identify relevant sources. Citation searching was also performed by screening the reference lists of included studies. No additional studies were sought by contacting authors or experts.

### Search

An initial limited search was conducted in PubMed/MEDLINE and Embase to identify relevant articles. Text words appearing in the titles, abstracts, and indexed descriptors of the retrieved studies were analyzed to inform the development of a comprehensive search strategy. Using the refined terms, a full search was subsequently performed in PubMed/MEDLINE, Embase, Scopus, and Web of Science. The reference lists of included studies were manually screened to identify additional relevant sources of evidence.

The search strategy was also informed by the Medical Subject Headings (MeSH) vocabulary [27], which was consulted to identify relevant concepts and synonyms. Controlled vocabulary, free-text terms, and field modifiers were combined using Boolean operators (AND/OR), with search strategies adapted to the specific characteristics of each database in accordance with methodological guidelines for systematic and scoping reviews [24].

PubMed/MEDLINE supports comprehensive free-text searching across multiple fields and uses Automatic Term Mapping to map search terms to MeSH, synonyms, and other relevant terms when applicable. Given its central role in indexing biomedical and public health literature, a high-sensitivity search strategy was implemented in PubMed/MEDLINE. The search was conducted using the “All Fields” option in combination with the species: humans filter to maximize retrieval, consistent with the exploratory nature of scoping review methodology.

In contrast, databases with different indexing structures required structured field restrictions to optimize retrieval precision and minimize nonrelevant records. Accordingly, searches in Scopus were limited to TITLE-ABS-KEY fields, while in Web of Science the Topic (TS) field was used, encompassing titles, abstracts, and author keywords. In Embase, database-specific syntax was applied, combining controlled vocabulary (Emtree terms with explosion, where appropriate) and free-text terms restricted to title, abstract, and keyword fields (ti, ab, and kw).

These adaptations reflect inherent differences in indexing systems and search functionalities across databases. The search was not restricted by publication date or study design to ensure comprehensive and exploratory coverage of the available evidence, consistent with the objectives of a scoping review. Language restrictions (English, Spanish, and Portuguese) were applied where available using database filters and were also considered during the eligibility assessment, reflecting the language proficiency of the review team, ensuring accurate interpretation of the included evidence, and aligning with the scope of the review as defined by the eligibility criteria. A filter for studies involving humans was applied where available.

The full search strategies for all databases are provided in Multimedia Appendix 2. Additionally, the PRISMA-S (Preferred Reporting Items for Systematic Reviews and Meta-Analyses Literature Search Extension) checklist [28] is provided in Multimedia Appendix 3 to enhance transparency and completeness in reporting the search strategy and information sources.

### Selection of Sources of Evidence

After applying the search strategy across the 4 selected databases, all records were imported into Rayyan (Rayyan Systems Inc), where potential duplicates were flagged using its automatic deduplication function, and all duplicate removals were confirmed through manual review before exclusion. Titles and abstracts were then screened according to predefined inclusion and exclusion criteria. Full texts of potentially eligible studies were subsequently assessed for eligibility. Screening was conducted by 2 independent reviewers, and any conflicts regarding study eligibility were resolved by a third reviewer. All selected sources were reviewed by an expert in the field of RDs. The search results and sources of evidence selection process were reported in accordance with the PRISMA 2020 flow diagram [25], allowing the presentation of the number of sources of evidence included at each stage of the review. The reasons for excluding each article were duly documented.

### Data Charting Process

Data were charted from the included sources of evidence using a structured spreadsheet developed by the research team. Data charting was performed by 1 reviewer and verified by a second reviewer. Discrepancies were resolved through discussion. No attempts were made to contact study authors for additional information.

### Data Items

The extracted data included authors, title, year of publication, origin/geographic context, study purposes, methods, and key findings for each included source of evidence. The complete dataset is provided in Multimedia Appendix 4.

### Critical Appraisal of Individual Sources of Evidence

A formal critical appraisal of the included sources of evidence was not conducted, as the objective of this scoping review was to map and synthesize the available literature rather than assess methodological quality.

### Synthesis of Results

The findings from the included sources of evidence were organized into thematic categories based on similarities identified during the data extraction process and through consensus among the authors. Each category consisted of specific elements reported across the included sources. Descriptive statistics were used to summarize the frequency and proportion of outcome measures, allowing comparison of patterns and convergence of information across different health care contexts.

This approach facilitated the synthesis and organization of information through tabulation. Based on these categories, we calculated the proportions of sources of evidence that indicated convergence with the established recommendations. The organized data were then analyzed and interpreted, considering that each selected source of evidence could have mentioned elements across multiple categories. The resulting thematic categories and their respective components are presented in Table 1.

**Table 1:** Thematic categories and their components identified in the included sources of evidence.

Category	Key points
Professional education and training in RD^a^	Knowledge gaps among health care professionals regarding RDs Lack of continuous education and technical training Inclusion of RD topics in health care curricula within undergraduate and postgraduate programs Organization of workshops, seminars, courses, and training sessions based on up-to-date evidence Implementation of continuing education initiatives Encouragement of the involvement of subject-matter experts in the educational process to enhance academic and professional training
Use of digital technologies and online platforms	Implementation of digital health functionalities in health care processes related to RDs Development and maintenance of digital platforms as repositories of validated evidence for consultation Strategic use of social media for communication, reducing information inequality, and promoting health and awareness Strengthening records in health information systems
Public policies, government actions, and cross-sectoral integration	Development of effective public policies for RDs Government investment and funding for health education programs Integration between the health and education sectors Development of strategies at regional, international, and global levels Creation of research networks and standardization of procedures Use and improvement of management and health indicators
Education and awareness for the general population	Promotion of educational initiatives for patients, families, and associations Dissemination of validated public health information to the general population Distribution of content through the internet and printed informational materials Use of clear, accessible, and transparent language
Emotional support and sharing of experiences	Promotion of emotional support among professionals, patients, families, and associations Sharing of experiences among professionals, patients, families, and associations as a form of empowerment
Awareness events and commemorative dates	Organization and implementation of public events and campaigns Establishment of specific dates to raise awareness for the RDs cause Awareness-raising actions for social engagement

^a^RD: rare disease.

## Results

### Selection of Sources of Evidence

Initially, a total of 3432 records were identified across 4 selected databases (PubMed/MEDLINE, n=1348; Embase, n=1643; Scopus, n=351; Web of Science, n=90). After removing 465 duplicates, a total of 2967 records remained for title and abstract screening. Of these, 221 full-text articles were assessed for eligibility. Following full-text review, 119 studies were excluded for not addressing the research question or for lacking the scientific rigor required to meet the objectives of this investigation, and 71 were excluded for being applied to specific clinical contexts or specific RD subgroups without addressing broader educational or awareness-related aspects. Hence, 31 studies were included in the review from the databases.

Regarding gray literature, 50 records were initially identified, including 35 from websites and 15 from documents produced by institutional organizations. Additionally, citation searching identified 28 records. In total, 78 records were identified through other methods. All records were screened and assessed for eligibility according to the predefined criteria.

**Figure 1:**
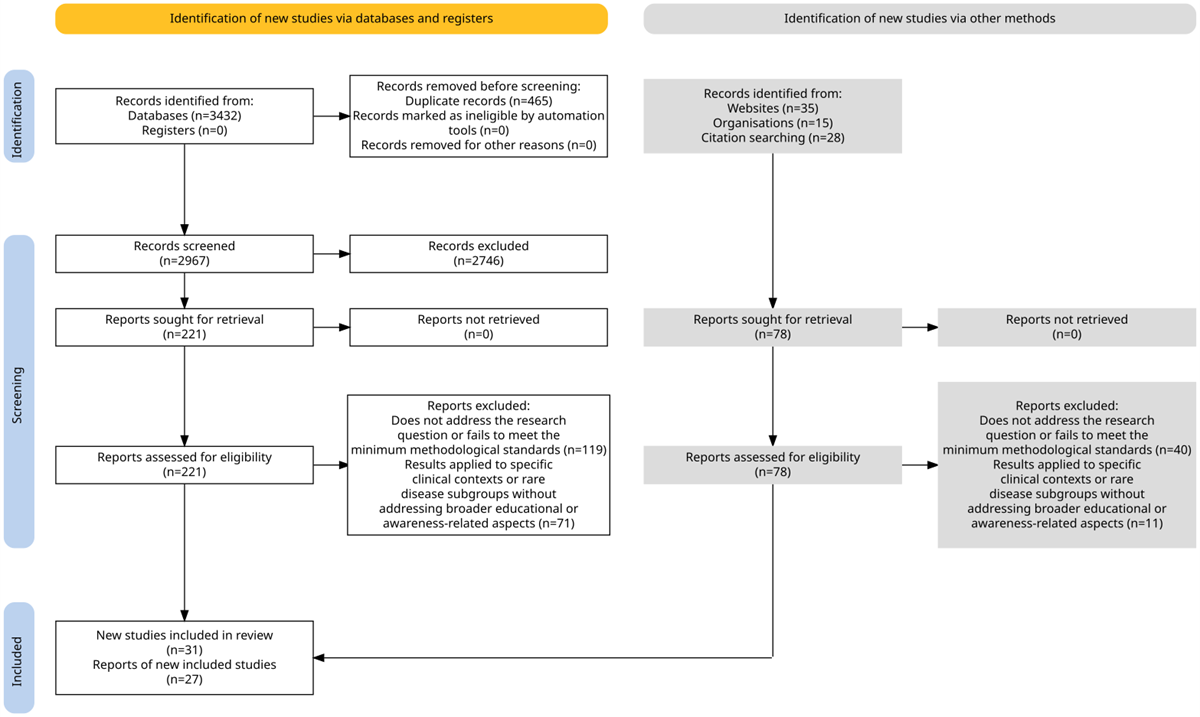
PRISMA (Preferred Reporting Items for Systematic Reviews and Meta-Analyses) 2020 flow diagram.

Among these 78 records, 40 records were excluded because they did not address the research question or failed to meet the minimum methodological standards, and 11 were excluded because their results applied to specific clinical contexts or RD subgroups without addressing broader educational or awareness-related aspects. As a result, 27 sources of evidence identified through other methods were included. In total, 58 sources of evidence were included in the scoping review (Multimedia Appendix 4 provides the complete list of included sources). The selection process is illustrated in Figure 1.

### Characteristics of Sources of Evidence

After selecting the sources of evidence included in the scoping review, relevant data were extracted to address the research question and systematically organize the main health education and awareness recommendations within the scope of RDs. The general characteristics of the included sources of evidence are presented in Multimedia Appendix 4.

### Critical Appraisal Within Sources of Evidence

No results of critical appraisal are reported because no formal critical appraisal of the included sources of evidence was conducted.

### Results of Individual Sources of Evidence

The detailed outcomes reported by each included source of evidence are presented in Multimedia Appendix 4, which summarizes the main results extracted from the included records.

### Synthesis of Results

Of the 58 selected sources of evidence, and considering that a single record could report information relevant to more than 1 category, professional education and training in RDs was the most frequently reported recommendation (49/58, 84.4%), followed by public policies, government actions, and cross-sectoral integration (36/58, 62%), education and awareness for the general population (28/58, 48.2%), the use of digital technologies and online platforms (27/58, 46.5%), emotional support and experience sharing (20/58, 34.4%), and awareness events and commemorative dates (8/58, 13.7%). Percentages are not mutually exclusive.

**Figure 2:**
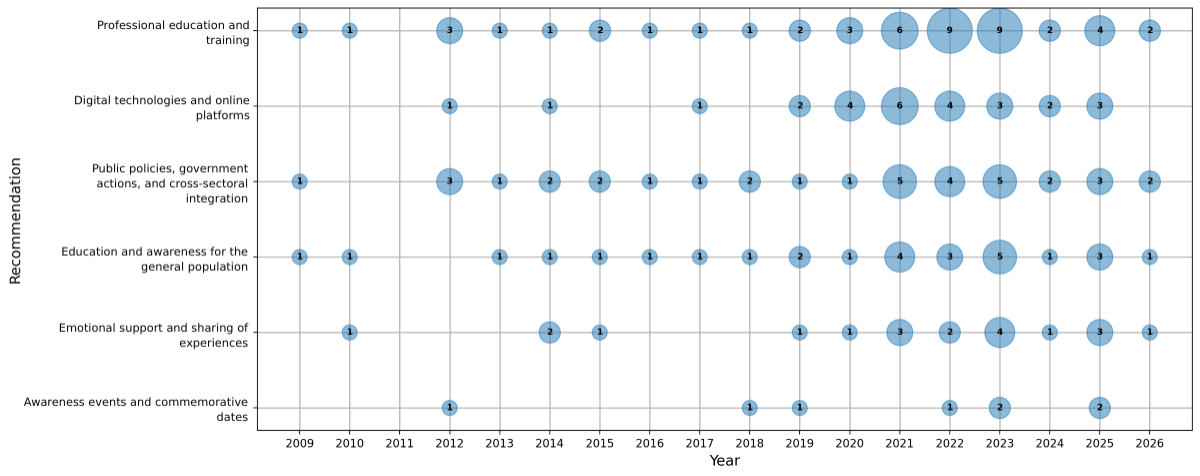
Temporal distribution of recommendations identified in the included sources of evidence.

As presented in Figure 2, there is a clear increase in the frequency of recommendations over time, particularly in the categories of professional education and training and digital technologies and online platforms, especially after 2019. Public policies, government actions, and cross-sectoral integration also show a consistent presence across the years, with moderate growth in recent periods. Recommendations related to education and awareness for the general population appear with a more irregular distribution, while those related to emotional support and experience sharing and awareness events and commemorative dates occur less frequently compared with the other recommendations.

Regarding the geographic distribution of this body of work, approximately 67.2% (39/58) of the sources of evidence were conducted at the country level. Examples include sources conducted in Poland [29-32], Germany [33-35], Australia [36-40], United States [41-43], Belgium [44], Canada [45], China [46-49], Brazil [50,51], Spain [52,53], Tanzania [54], Mexico [55], Bahrain [56], Bulgaria [57], Iran [58], India [59], and Kazakhstan [60]. Other sources addressed broader geographical regions, including Europe [61-67], Latin America [68], Asia-Pacific [69], Southern Africa [70], North America [71], as well as globally oriented records [16,72-78]. A comprehensive view of the geographic distribution of the included sources is presented in Table 2.

**Table 2:** Distribution of sources of evidence by country and region.

Country/region	Sources, n
Global	8
Multicountry (Europe)	7
Poland	5
Australia	5
China	4
Germany	3
United States	3
Brazil	2
Spain	2
Turkey	2
Peru	2
United Kingdom	1
Kazakhstan	1
Tanzania	1
Belgium	1
Mexico	1
Bahrain	1
Multicountry (Latin America)	1
Multicountry (Asia-Pacific)	1
Multicountry (Southern Africa)	1
Bulgaria	1
Canada	1
Iran	1
India	1
Serbia	1
Multicountry (North America)	1

**Figure 3:**
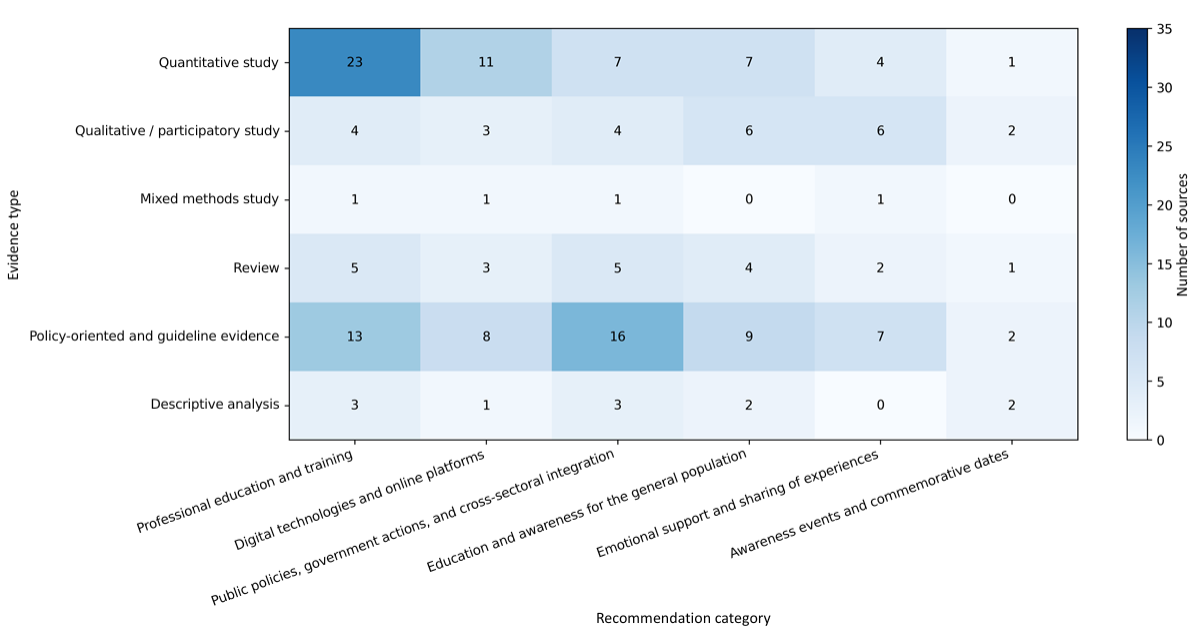
Distribution of recommendation categories across evidence types.

A higher concentration was observed in globally oriented and multicountry sources in Europe, while individual countries such as Poland, Australia, China, Germany, and the United States contributed substantially. Conversely, the presence of numerous countries and regions represented by only 1 or 2 sources suggests a fragmented geographic distribution of the evidence. Additionally, to facilitate synthesis and interpretation of the findings, study designs were aggregated into broader evidence-type categories, and their distribution across recommendation categories is presented in Figure 3.

Quantitative studies were frequently represented across several recommendation categories, particularly in professional education and training, which showed the highest number of quantitative sources and demonstrated relatively broad methodological diversity. These findings suggest that professional education and training are among the most consolidated areas within the literature and that the training and capacity building of health care professionals have been widely prioritized as a central strategy in the context of RDs.

Recommendations related to public policies, government actions, and cross-sectoral integration were strongly associated with policy-oriented and guideline evidence, indicating substantial involvement of institutional and governmental actors in shaping recommendations in this area. Recommendations related to emotional support and experience sharing showed a meaningful contribution from qualitative and participatory studies, reflecting the relevance of patient-centered and experience-based approaches to understanding the social and emotional dimensions associated with RDs. Policy-oriented and guideline evidence, however, was also frequently represented within this category.

**Figure 4:**
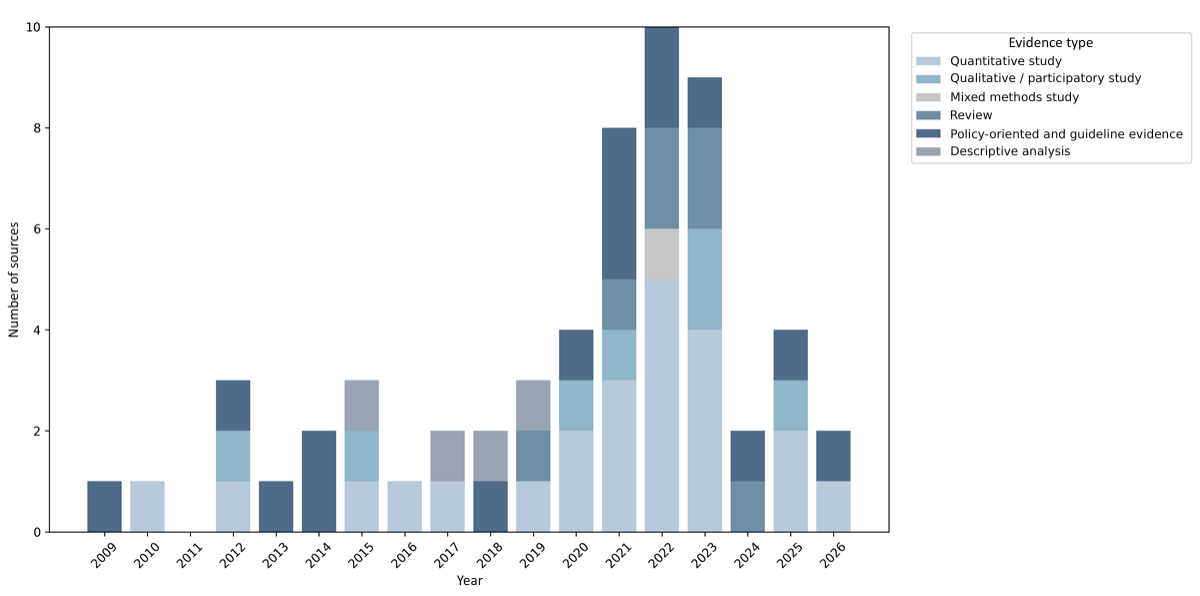
Temporal evolution of evidence types identified in the included sources of evidence.

A generally low representation of mixed methods studies was observed across all recommendation categories, suggesting limited integration between methodological approaches and highlighting a potential gap in the field. Awareness events and commemorative dates represented the least frequently reported category across nearly all evidence types, which may indicate comparatively lower attention to campaign-based strategies within the literature. Meanwhile, recommendations related to digital technologies and online platforms demonstrated a heterogeneous methodological distribution, encompassing quantitative, qualitative, review-based, as well as policy-oriented and guideline evidence, suggesting growing multidisciplinary interest in the use of digital tools to support education and awareness strategies for RDs. Overall, the coexistence of diverse types of evidence reinforces the multidimensional and interdisciplinary nature of health education and awareness strategies in the context of RDs. The temporal distribution of evidence types across the included sources is illustrated in Figure 4.

A progressive increase in evidence production over time was observed, particularly from 2019 onward, with a peak between 2021 and 2023. Quantitative studies represented the predominant evidence type throughout most of the temporal series, especially in the most recent period. Policy-oriented and guideline evidence, as well as qualitative and participatory studies, became more frequently represented after 2020. Overall, the findings indicate a gradual diversification of evidence types over time, with recent years showing broader methodological diversity and greater coexistence of quantitative, qualitative, review-based, and policy-oriented and guideline evidence.

Concerning the thematic content of the recommendations, many sources highlighted a lack of structured knowledge about RDs among health care professionals and students across various roles, as well as other informational gaps related to insufficient training, capacity building, and continuing education opportunities for these professionals [33,35,40,50,56,58,59,79,80]. It is therefore recommended that curricular content on RDs be strengthened and expanded within undergraduate and postgraduate programs [29,30,32,43,46,50,55,58,71].

In addition, continuing education initiatives, including targeted training and capacity-building courses for practicing professionals, should be implemented, and workshops grounded in up-to-date scientific evidence and clinical guidelines should also be regularly offered to support continuous knowledge development [16,18,36,60,62,65,69,70,76]. Furthermore, the involvement of subject-matter experts in this educational process is encouraged to enhance academic and professional training [57,61].

Another issue raised was the need to leverage digital health functionalities to support RD processes, as well as the development, maintenance, and use of digital platforms and websites as repositories of validated evidence and knowledge for consultation purposes, in addition to strengthening records in health information systems, while internet communication channels and social media were also highlighted as active tools that can be used by health agencies and councils to address gaps in knowledge and access to information and to raise awareness about the topic [18,34,36-38,53,65,68,79].

The recommendations also emphasize the development of effective public health policies, increased government funding, closer integration between the health and education sectors, and coordinated regional, international, and global strategies [18,36,37,46,47,54,64,67,74]. Additionally, they highlight the creation of research networks and committees, the standardization of procedures, the improvement of indicators and system records, and the definition of best practices in management, organization, and innovation [51,54,55,61,62,69,72,74,81].

The need for education and awareness initiatives on RDs for the general population was also mentioned, emphasizing the dissemination of validated and relevant information to patients, families, and associations through educational actions and programs, including making such content available and distributing educational and informational materials in different formats in a clear and accessible way [35,42,46,63-65,67,74,82].

Emotional and psychological support, as well as the sharing of experiences among health care professionals, patients, families, and associations, were also identified as important factors in this scenario [16,34,35,40,42,45,51,53,75]. In addition, the organization of awareness events and the establishment of specific dates were highlighted as strategies to strengthen the RDs agenda and draw the attention of society to this public health issue [16,18,38,49,53,55,56,75].

## Discussion

### Summary of Evidence

The main findings of this scoping review indicate that recommendations for developing educational and awareness strategies in the context of RDs focus on professional education and training; use of digital technologies and online platforms; public policies, government actions, and cross-sectoral integration; education and awareness for the general population; emotional support and sharing of experiences; and awareness events and commemorative dates. In line with the objective of identifying and systematizing the main recommendations for health education and awareness in the context of RDs, the evidence suggests that improving knowledge dissemination and awareness in this field requires integrated approaches that combine clinical education, accessible information systems, institutional coordination, and patient-centered support strategies. Collectively, these dimensions reinforce that education and awareness in RDs extend beyond information delivery alone and depend on coordinated actions involving health care systems, governments, educational institutions, patient organizations, and society.

The evidence synthesized in this scoping review indicates that education and awareness processes related to RDs remain fragmented and insufficiently structured in many health care settings, creating significant barriers to diagnosis, management, and continuity of care. Several included sources highlighted persistent knowledge gaps among health care professionals and medical students, particularly regarding the recognition, diagnosis, and management of these conditions [29,30,32,35,43,44,46-48,50,52,56-60,62,71,80,83,84]. These findings reflect broader structural limitations within professional training programs, where RD-related content is often underrepresented in undergraduate and continuing education curricula. Consequently, physicians, nurses, and students frequently report low confidence in their ability to identify and manage RDs, compromising timely diagnosis and coordinated care [29,50,52,60]. These findings support previous recommendations emphasizing the need for systematic educational reforms and continuous professional development initiatives specifically tailored to RDs [16,36,37,45,50,54,64,68,70,75].

Within this context, our findings also highlight the growing importance of digital technologies and online knowledge platforms as tools to support education, diagnostic processes, and access to information related to RDs [18,37,40,44,79]. The lack of accessible and reliable information remains one of the major barriers to early and accurate diagnosis [16,55,63,68,69,79], and digital resources have increasingly emerged as valuable tools to address this gap. Structured platforms and ontologies such as the Human Phenotype Ontology, the Orphanet Rare Disease Ontology, and PubCaseFinder exemplify this movement by enabling standardized phenotypic descriptions, facilitating symptom-based searches, and supporting diagnostic processes through evidence-based systems [85-87]. Beyond their clinical applications, these resources have progressively been incorporated into educational settings, helping health care professionals and students improve the structured description and interpretation of phenotypes and supporting diagnostic investigation [88-90].

The use of online educational resources also appears to strengthen information dissemination and patient support. Sources included in this scoping review emphasize the relevance of websites, digital repositories, and online educational materials as accessible resources for patients, caregivers, and professionals [34,38,48,77]. In many situations, online information platforms initially represent the primary source of knowledge for patients and families before they establish contact with specialists or support organizations [34]. These findings are consistent with broader global trends in digital health and reinforce the importance of developing accessible, validated, and user-centered information systems capable of supporting both educational processes and clinical decision-making [91].

Another important dimension identified in this study involves the role of public policies, institutional coordination, and cross-sectoral collaboration in strengthening RD education and awareness strategies. The included sources consistently emphasize the importance of improving patient registries, fostering collaborative networks, funding research, and standardizing clinical and managerial processes [40,51,61-63,66,69]. These actions are aligned with international recommendations proposed by the WHO, which advocate for comprehensive and integrated policies intended to improve access to care, monitor health care indicators, and strengthen national and regional health systems for RDs [92]. In addition, national and international initiatives have highlighted the importance of communication strategies that promote accessible and inclusive health information tailored to different audiences, including patients, families, health care professionals, and the general population [34,35,41,65].

The findings further suggest that awareness and educational efforts in the context of RDs should not be restricted to clinical or institutional dimensions alone. Emotional and psychological support emerged as essential components for improving quality of life and strengthening care pathways for patients and families affected by RDs [18,35,40,53,73,82]. In this context, experience sharing among patients, caregivers, and health care professionals plays a fundamental role in building support networks, reducing social isolation, and fostering resilience. Social media platforms such as YouTube, Facebook, and Instagram have increasingly become important spaces for information exchange, peer support, and patient engagement, particularly for individuals facing prolonged diagnostic journeys and limited access to specialized services [93-95].

The integration of patient support groups and participatory educational strategies also appears to strengthen patient empowerment and improve the relevance of communication initiatives. Previous studies emphasize that health care professionals can contribute to these processes by moderating discussions, supporting the dissemination of reliable information, and facilitating the referral of newly diagnosed patients to support communities [96,97]. Furthermore, participatory approaches involving patients and caregivers in the codevelopment of educational resources and awareness strategies may enhance communication effectiveness and ensure that educational initiatives better reflect the lived experiences and practical needs of individuals affected by RDs [45]. These findings reinforce the importance of adopting collaborative and patient-centered models in RD education and awareness initiatives.

Finally, this scoping review highlights the relevance of awareness events, campaigns, and commemorative dates in increasing the visibility of RDs and promoting broader societal engagement [18,37,38,49,54,55]. Public awareness actions, including initiatives such as Rare Disease Day, represent important opportunities to mobilize policymakers, health care institutions, educational organizations, patient associations, and the general public around the challenges associated with RDs. In parallel, collaboration between health and education systems may contribute to the development of more sustainable and coordinated awareness strategies, capable of integrating scientific knowledge, professional training, public engagement, and patient advocacy efforts [18,37].

### Limitations

Among the limitations of this study, the variability in the territorial scope of the included sources may limit the generalizability of the findings to settings with different income levels or less structured health care systems. In addition, differences were identified in study designs, data sources, methodological approaches, and outcome measures (Multimedia Appendix 4 provides full details). These factors may influence the interpretation and comparability of findings and should therefore be considered when applying the results of this scoping review.

Nevertheless, many of the principles identified, such as the need for intersectoral policies, government actions, professional education and capacity building, emotional support and the sharing of experiences, and increased education and awareness among the general population, are consistent with global guidelines, including those proposed by the WHO for health care management [16,91]. However, the feasibility and implementation of some of the identified recommendation categories may vary across resource and income settings and may require contextual adaptation according to regional and institutional characteristics. In particular, the use of digital technologies and online platforms depends on the availability of appropriate infrastructure and may require adaptation in resource-constrained settings [2,16,46,91].

Similarly, integration across referral networks, specialized centers, and cross-sectoral systems may pose additional implementation challenges [98]. Professional education and training are widely recognized as key strategies across different contexts but may vary in effectiveness depending on how well they are designed and delivered to meet the needs of different target audiences [29,44,46,50,77]. In addition, recommendations related to public awareness, emotional support, experience sharing, and awareness events are widely reported and implemented in a variety of contexts [34,41,45,49,65,66,68].

Additionally, although a formal assessment of methodological quality among the included sources is not standard practice in scoping reviews, we acknowledge that the robustness of the included evidence varies across sources. In this context, future research could build upon the evidence mapped in this study by conducting approaches such as systematic reviews that critically assess the methodological quality of included sources, using appraisal tools such as the Critical Appraisal Skills Programme [99] or A Measurement Tool to Assess Systematic Reviews-2 [100].

The findings presented in this study may therefore serve as a preliminary foundation for further exploration in this field. Furthermore, the language restriction applied during the selection process may have limited access to relevant sources published in excluded languages, particularly those originating from Eastern regions or from countries underrepresented in international databases. However, as much of the available evidence is commonly disseminated in English and indexed in major databases, the impact of this limitation may be partially mitigated.

Despite these limitations, the findings of this study contribute to expanding the current body of evidence on recommendations for educational and awareness strategies related to RDs, underscoring the relevance of integrated, multisectoral approaches. By highlighting the intersection between health, education, communication, policy, and society, this review reinforces the need to engage diverse social actors in the design and implementation of effective and context-sensitive initiatives. Ultimately, strengthening such collaborative efforts may support improved knowledge dissemination, earlier recognition, more inclusive care pathways, and more informed health planning and decision-making in the complex context of RDs.

### Conclusions

This scoping review mapped and synthesized existing recommendations related to the development of health education and awareness strategies for RDs. The findings contribute to understanding how these strategies have been addressed in the literature, highlighting important gaps and variations across different contexts. They may support evidence-informed decision-making and public health planning, particularly regarding persistent bottlenecks in diagnostic processes and care coordination. Given the complexity of RD care, the scarcity of available knowledge associated with these conditions, and their substantial societal impact, strengthening health education and awareness strategies may contribute to broader public health efforts aimed at improving access to information, continuity of care, and health care management in the context of RDs.

Future research should explore the effectiveness and implementation of specific health education and awareness strategies for RDs across different contexts. More focused systematic reviews may also assess the impact of particular interventions, building upon the body of evidence identified in this scoping review while critically evaluating the methodological quality of included sources. Overall, by consolidating dispersed and heterogeneous evidence into a coherent body of knowledge, this scoping review contributes to advancing the field and may inform improvements in health services, professional and managerial practices, and initiatives aimed at supporting patients, families, and advocacy groups involved in RDs.

## Data Availability

Data generated or analyzed during this study are included in this published article and its supplementary information files. Additional datasets generated or analyzed during this study are available from the corresponding author on reasonable request.

## Appendix

Multimedia Appendix 1 PRISMA-ScR checklist.

Multimedia Appendix 2 Full search strategies for databases.

Multimedia Appendix 3 PRISMA-S checklist.

Multimedia Appendix 4 The general characteristics of the included sources.

## Abbreviations

MeSH: Medical Subject Headings
PCC: Population, Concept, and Context
PRISMA: Preferred Reporting Items for Systematic Reviews and Meta-Analyses
PRISMA-S: Preferred Reporting Items for Systematic Reviews and Meta-Analyses Literature Search Extension
PRISMA-ScR: Preferred Reporting Items for Systematic Reviews and Meta-Analyses Extension for Scoping Reviews
RD: rare disease
WHO: World Health Organization
