# Supplementary material for: Main Recommendations for Developing Education and Awareness Strategies for Rare Diseases: Scoping Review
Source: JMIR Med Educ. 2026 Jun 12;12:e79027. doi: 10.2196/79027 (PMC13309765; doi:10.2196/79027)
Supplement: Multimedia Appendix 2 [file mededu_v12i1e79027_app2.docx]

| Database | Search strategy |
| --- | --- |
|  |  |
| PubMed/MEDLINE | ("Health Education"[Mesh] OR "Health Literacy"[Mesh] OR "Health Promotion"[Mesh] OR "Patient Education as Topic"[Mesh] OR health education OR health awareness OR health literacy OR patient education [tiab] OR disease education [tiab] OR health communication [tiab] OR health knowledge [tiab] OR knowledge dissemination [tiab] OR health information [tiab] OR information dissemination [tiab] OR health promotion [tiab]) AND ("Rare Diseases"[Mesh] OR rare disease* OR orphan disease* OR rare disorder* OR rare condition* OR orphan disorder* OR orphan condition* [tiab] OR rare genetic disease* [tiab] OR inherited rare disease* [tiab]) AND (recommendation* [tiab] OR clinical recommendation* [tiab] OR management recommendation* [tiab] OR practice recommendation* [tiab] OR consensus recommendation* [tiab] OR guideline* [tiab] OR practice guideline* [tiab] OR consensus statement* [tiab] OR position statement* [tiab] OR policy* [tiab] OR guidance [tiab] OR best practice* [tiab] OR practice standard* [tiab] OR "Practice Guideline"[Publication Type] OR "Guidelines as Topic"[Mesh]) |
| Scopus | ("health education" OR "health awareness" OR "health literacy" OR "patient education" OR "disease education" OR "health communication" OR "health knowledge" OR "knowledge dissemination" OR "health information" OR "information dissemination" OR "health promotion" ) AND ( "rare disease*" OR "orphan disease*" OR "rare disorder*" OR "rare condition*" OR "orphan disorder*" OR "orphan condition*" OR "rare genetic disease*" OR "inherited rare disease*" ) AND ( "recommendation*" OR "clinical recommendation*" OR "management recommendation*" OR "practice recommendation*" OR "consensus recommendation*" OR "guideline*" OR "practice guideline*" OR "consensus statement*" OR "position statement*" OR "policy*" OR "guidance" OR "best practice*" OR "practice standard*") |
| Web of Science | ("health education" OR "health awareness" OR "health literacy" OR "patient education" OR "disease education" OR "health communication" OR "health knowledge" OR "knowledge dissemination" OR "health information" OR "information dissemination" OR "health promotion") AND ("rare disease*" OR "orphan disease*" OR "rare disorder*" OR "rare condition*" OR "orphan disorder*" OR "orphan condition*" OR "rare genetic disease*" OR "inherited rare disease*") AND ("recommendation*" OR "clinical recommendation*" OR "management recommendation*" OR "practice recommendation*" OR "consensus recommendation*" OR "guideline*" OR "practice guideline*" OR "consensus statement*" OR "position statement*" OR "policy*" OR "guidance" OR "best practice*" OR "practice standard*") |
| Embase | ('health education'/exp OR 'health education':ti,ab,kw OR 'health literacy'/exp OR 'health literacy':ti,ab,kw OR 'health awareness':ti,ab,kw OR 'patient education'/exp OR 'patient education':ti,ab,kw OR 'disease education':ti,ab,kw OR 'health communication':ti,ab,kw OR 'health knowledge':ti,ab,kw OR 'knowledge dissemination':ti,ab,kw OR 'health information':ti,ab,kw OR 'information dissemination':ti,ab,kw OR 'health promotion':ti,ab,kw) AND ('rare disease'/exp OR 'rare disease*':ti,ab,kw OR 'orphan disease*':ti,ab,kw OR 'rare disorder*':ti,ab,kw OR 'rare condition*':ti,ab,kw OR 'orphan disorder*':ti,ab,kw OR 'orphan condition*':ti,ab,kw OR 'rare genetic disease*':ti,ab,kw OR 'inherited rare disease*':ti,ab,kw) AND ('recommendation*':ti,ab,kw OR 'clinical recommendation*':ti,ab,kw OR 'management recommendation*':ti,ab,kw OR 'practice recommendation*':ti,ab,kw OR 'consensus recommendation*':ti,ab,kw OR 'guideline'/exp OR 'guideline*':ti,ab,kw OR 'practice guideline'/exp OR 'practice guideline*':ti,ab,kw OR 'consensus statement*':ti,ab,kw OR 'position statement*':ti,ab,kw OR 'policy*':ti,ab,kw OR 'guidance':ti,ab,kw OR 'best practice':ti,ab,kw OR 'practice standard':ti,ab,kw) AND ([english]/lim OR [portuguese]/lim OR [spanish]/lim) |
